# Lessons Learned in Conducting Mass Drug Administration for Schistosomiasis Control and Measuring Coverage in an Operational Research Setting

**DOI:** 10.4269/ajtmh.19-0789

**Published:** 2020-05-12

**Authors:** Sue Binder, Carl H. Campbell, Jennifer D. Castleman, Nupur Kittur, Safari M. Kinung’hi, Annette Olsen, Pascal Magnussen, Diana M. S. Karanja, Pauline N. M. Mwinzi, Susan P. Montgomery, William Evan Secor, Anna E. Phillips, Neerav Dhanani, Pedro H. Gazzinelli-Guimaraes, Michelle N. Clements, Eliézer K. N’Goran, Aboulaye Meite, Jürg Utzinger, Amina A. Hamidou, Amadou Garba, Fiona M. Fleming, Christopher C. Whalen, Charles H. King, Daniel G. Colley

**Affiliations:** 1 Schistosomiasis Consortium for Operational Research and Evaluation, Center for Tropical and Emerging Global Diseases, University of Georgia, Athens, Georgia;; 2 Mwanza Research Centre, National Institute for Medical Research, Mwanza, Tanzania;; 3 Section for Parasitology and Aquatic Pathobiology, Faculty of Health and Medical Sciences, University of Copenhagen, Copenhagen, Denmark;; 4 Centre for Medical Parasitology, Faculty of Health and Medical Sciences, University of Copenhagen, Copenhagen, Denmark;; 5 Centre for Global Health Research, Kenya Medical Research Institute, Kisumu, Kenya;; 6 Division of Parasitic Diseases and Malaria, Centers for Disease Control and Prevention, Atlanta, Georgia;; 7 Schistosomiasis Control Initiative, Imperial College, London, United Kingdom;; 8 Unité de Formation et de Recherche Biosciences, Université Félix Houphouët-Boigny, Abidjan, Côte d’Ivoire;; 9 Centre Suisse de Recherches Scientifiques en Côte d’Ivoire, Abidjan, Côte d’Ivoire;; 10 Programme National de Lutte contre les Maladies Tropicales Négligées à Chimiothérapie Préventive, Abidjan, Côte d’Ivoire;; 11 Swiss Tropical and Public Health Institute, Basel, Switzerland;; 12 University of Basel, Basel, Switzerland;; 13 Réseau International Schistosomoses, Environnement, Aménagement et Lutte (RISEAL-Niger), Niamey, Niger;; 14 Department of Control of Neglected Tropical Diseases, Preventive Chemotherapy and Transmission Control Unit, World Health Organization, Geneva, Switzerland;; 15 Department of Epidemiology and Biostatistics, Global Health Institute, University of Georgia, Athens, Georgia;; 16 Center for Global Health and Diseases, Case Western Reserve University, Cleveland, Ohio;; 17 Department of Microbiology, University of Georgia, Athens, Georgia

## Abstract

The Schistosomiasis Consortium for Operational Research and Evaluation (SCORE) was created to conduct research that could inform programmatic decision-making related to schistosomiasis. SCORE included several large cluster randomized field studies involving mass drug administration (MDA) with praziquantel. The largest of these were studies of gaining or sustaining control of schistosomiasis, which were conducted in five African countries. To enhance relevance for routine practice, the MDA in these studies was coordinated by or closely aligned with national neglected tropical disease (NTD) control programs. The study protocol set minimum targets of at least 90% for coverage among children enrolled in schools and 75% for all school-age children. Over the 4 years of intervention, an estimated 3.5 million treatments were administered to study communities. By year 4, the median village coverage was at or above targets in all studies except that in Mozambique. However, there was often a wide variation behind these summary statistics, and all studies had several villages with very low or high coverage. In studies where coverage was estimated by comparing the number of people treated with the number eligible for treatment, denominator estimation was often problematic. The SCORE experiences in conducting these studies provide lessons for future efforts that attempt to implement strong research designs in real-world contexts. They also have potential applicability to country MDA campaigns against schistosomiasis and other NTDs, most of which are conducted with less logistical and financial support than was available for the SCORE study efforts.

## INTRODUCTION

Preventive chemotherapy through mass drug administration (MDA) with praziquantel (PZQ) is the current mainstay of the global strategy to control schistosomiasis.^1–3^ According to the World Health Organization (WHO), successful implementation of MDA for schistosomiasis control is defined as treating at least 75% of the eligible at-risk school-age children (SAC) within an affected community.^4^ Problems with both measuring MDA coverage and achieving targets have been reported previously.^5–7^

The Schistosomiasis Consortium for Operational Research and Evaluation (SCORE) was launched in 2008 with funding from the Bill & Melinda Gates Foundation to contribute to the evidence base for programmatic decision-making related to schistosomiasis control and elimination. The SCORE research portfolio includes studies related to topics such as control and elimination of schistosomiasis, diagnostic test development and validation in real-world settings, and changes in schistosome population genetics in response to drug pressure.^8^

Several SCORE studies involved conducting MDA with PZQ and measuring village-level coverage for each round of MDA. Coverage in the Zanzibar elimination study has been previously reported.^7^ This article focuses on coverage results and issues encountered during the largest of the SCORE intervention studies—the “gaining and sustaining control studies.”^9^ These studies involved evaluation of different MDA strategies for control of *Schistosoma haematobium* and *Schistosoma mansoni* infections in five African countries.^9^ A number of articles have been published describing the baseline characteristics of the study populations and some of the main study outcomes, including changes in infection prevalence and intensity among children aged 9–12 years after 4 years of intervention.^10–16^

To ensure that our findings would have relevance to ongoing schistosomiasis control and elimination efforts, the SCORE gaining and sustaining control studies were conducted in the context of country neglected tropical disease (NTD) programs, with MDA coordinated by or aligned with national NTD programs. However, the studies also involved aspects of field trial research, with random assignment of groups of people (i.e., villages) to different interventions. Our hope was that the involvement of national control programs would ensure the use of the findings within the country, whereas the inclusion of field trial research methods would contribute to quality, generalizability, and the use of the findings across countries. These two perspectives—working in ways that aligned with ongoing national control programs, but performing a formal field trial—resulted in many opportunities and challenges. We believe our experiences and lessons learned will be relevant for future operational research that will require high coverage rates and quality coverage measurement in contexts where total control of interventions and measurement of intervention success are aspirational but may prove difficult.

## METHODS

### Gaining and sustaining control and Niger study designs.

The design for the gaining and sustaining control studies has been described in detail elsewhere.^9^ In brief, gaining control studies were performed in communities with ≥ 25% *Schistosoma* prevalence in 13- to 14-year-old children, as determined in eligibility surveys conducted before study implementation. These studies each had six study arms, which provided varying combinations of community-wide treatment (CWT), school-based treatment (SBT), and drug holidays, which were defined as years without MDA with PZQ, although other drugs such as albendazole for treatment of soil-transmitted helminth infections may have been provided.

Sustaining control studies were performed in communities having 10–24% prevalence among 13- to 14-year-old children during eligibility surveys. The three study arms involved annual SBT or combinations of SBT and drug holidays.

Both studies involved cluster randomization at the village level, with each study arm including 25 villages. Thus, there were 150 villages in each gaining control study and 75 in each sustaining control study.

The harmonized gaining and sustaining control protocol was developed with extensive input from funded investigators and other experts and provided a high-level outline of planned study interventions and endpoint measurements. It required the assessment of baseline prevalence and infection intensity in 9- to 12-year-old schoolchildren, then four intervention years (MDA with PZQ or drug holidays), followed by a fifth-year post-intervention parasitologic survey, also among 9- to 12-year-old schoolchildren. Mass drug administration was either conducted by the host country’s national NTD control program or in close coordination with it. Coverage was measured during or shortly after each MDA.

Gaining control studies were conducted in Kenya, Mozambique, and Tanzania, whereas sustaining control studies were conducted in Côte d’Ivoire and Kenya. Niger originally was funded to conduct both gaining and sustaining control studies for *S. haematobium*. However, the study protocol regarding randomization was not followed in Niger, where villages were instead grouped by geographic proximity to one another. Rather than terminate the study in Niger, it was redesigned in year 3 to evaluate twice- versus once-a-year treatment with either SBT or CWT.^12^

Most of the gaining and sustaining control studies began in early 2011. The last to start was that in Côte d’Ivoire, where the study start was delayed because of civil unrest and security issues.

### Mass drug administration and treatment coverage as defined in the SCORE harmonized protocol.

In villages randomized to SBT, PZQ was mainly administered by trained teachers, except in the early years of the Kenya and Tanzania gaining control studies, when it was administered in community-based efforts by community drug distributors (CDDs). In some cases, SBT occurred primarily in schools but was augmented by CDDs. The CDDs typically were NTD program staff, other community health workers, or study program hires. The protocol required efforts to include both enrolled and non-enrolled SAC in SBT villages by using outreach to encourage nonschool-attending children to come to the school for treatment.

Community-wide treatment programs were to provide treatment to the entire eligible population in the study community (i.e., children younger than 5 years or less than 94 cm in height were explicitly excluded, whereas pregnant women were to be included).^3^ The protocol called for community sensitization before MDA to help increase coverage rates, both for SBT (e.g., radio announcements and mobilization through schools to encourage participation of non-attending SAC) and CWT (e.g., radio announcements, community meetings, flyers, village criers, television notices, and other efforts targeting all community members). However, specific approaches to worker training and community sensitization were left to individual studies so that investigators could take their local experience and other contextual issues into account.

The protocol emphasized the need for high-quality coverage data. Coverage was defined as the percentage of targeted individuals who received PZQ. By protocol, ingestion of PZQ was to be directly observed. The protocol required a census of all study villages in year 1 to determine the number of people eligible for treatment (i.e., the coverage denominator), but allowed other “high-quality” data to be used instead. Those providing PZQ kept records of names, age, gender, height, numbers of tablets provided, and any other relevant data. Further requirements for coverage measurement were not specified.

The harmonized protocol set coverage targets of at least 90% among school-enrolled children and 75% coverage of all SAC. In CWT villages, there was an additional target of 75% of the entire eligible population. If coverage after the first attempt at MDA was less than these targets, teams were to return to the village for additional “mop-up” efforts in an attempt to achieve the coverage targets.

### Data sources for reconstructing the SCORE experience.

We used records kept by different SCORE studies, published articles, and recollections of individual investigators to catalog SCORE experiences related to coverage. Structured forms and tables were used to capture key information. Among the records reviewed were annual reports submitted to the SCORE secretariat, midterm reviews of each study, SCORE annual meeting presentations, summaries of phone and Skype calls, and e-mail correspondence. Investigators at each study site were to verify the compiled information and provide information missing from SCORE secretariat records.

### Ethics statement.

In each of the studies summarized in this article, written informed consent was obtained from adults (including parents/legal guardians of children in the study) and assent was obtained from children younger than 18 years, except in places where village-level consent is the standard, in which case local requirements were met. Ethical review of research protocols was implemented by human subjects committees in each African country and by the institutional review board (IRB) of their respective northern partners, and the IRB of the University of Georgia (Athens, GA). Details can be found in the publication by Ezeamama et al.^9^

The trials were registered with the International Standard Randomized Controlled Trial registry under ISRCT numbers 99401114 (Côte d’Ivoire sustaining control study), 14849830 (Kenya sustaining control study), 16755535 (Kenya gaining control study), 95819193 (Tanzania gaining control study), 32045736 (Niger study), and 14117624 (Mozambique gaining control study).

## RESULTS

### Sources of coverage data.

Table 1 shows sources of data for estimating coverage numerators and denominators. Numerators were obtained from teacher records, augmented by CDD registries when children were treated outside of school settings. For the most part, children treated in schools were directly observed taking the drugs. However, in Tanzania, when food was not available, PZQ was sometimes sent home with children to be taken when they had access to food. Although CDDs in all studies were told to observe individuals taking pills, CDDs in some studies left pills to be taken later when not all members of a household were at home.^17^ An estimated 3.5 million treatments were administered in the context of the SCORE gaining and sustaining studies and the Niger study.

**Table 1:** Main sources of numerator and denominator data used by SCORE studies to estimate MDA coverage

SCORE study	Type of MDA/population assessed	Source of numerator data	Source of denominator data
Côte d’Ivoire sustaining control	SBT (SAC)	Teacher records in year 1, when all treatment occurred in schools, and teacher and CDD treatment records in subsequent years	Year-by-year Ministry of Health reports of village-level total population. Twenty-six percent of the population of each village was assumed to be SAC
Kenya sustaining control	SBT (SAC)	Teacher treatment records	School enrollment
Kenya gaining control	SBT (SAC)	Teacher treatment records	School enrollment
	CWT	Years 1 and 2: CDD registries	Years 1 and 2: CDD census, updated regularly
		Years 3 and 4:	Years 3 and 4:
		• SAC: teacher treatment records and CDD registries	• SAC: School enrollment
		• Non-SAC: CDD registries	• Non-SAC: CDD census, updated regularly
Mozambique gaining control	SBT (SAC)	Teacher treatment records, non-enrolled children were encouraged to come to the school for treatment	2011 census. Thirty percent of the population of each village was assumed to be SAC
	CWT	SAC: teacher treatment records and CDD registries	2011 census
		Non-SAC: CDD registries	
Niger	SBT (SAC)	Teacher treatment records	Annual village-level data provided by the Ministry of Health
		SAC: teacher treatment records and CDD registries	
	CWT	Non-SAC: CDD registries	
Tanzania gaining control	SBT (SAC)	Teacher treatment records	Census information collected from the village executive official’s records
	CWT	Years 1 and 2: CDD registries	
		Years 3–4:	
		SAC: teacher treatment records	
		Non-SAC: CDD registries	

CDD = community drug distributor; CWT = community-wide treatment; MDA = mass drug administration; SAC = school-age children; SBT = school-based treatment; SCORE = Schistosomiasis Consortium for Operational Research and Evaluation.

Different studies used different approaches for assessing denominators. For example, in places where enrollment in primary education is high, SAC estimates were sometimes based on school enrollment.^18^ In some places, CDDs or study staff conducted censuses. In others, the Ministry of Health provided annual estimates of the total population of each village and the percent of each village’s population believed to be SAC. Tanzania has a robust system for routine census data collection; the estimates of total population and SAC eligible for treatment in both SBT and CWT villages were based on this village-level data collection.

### Coverage estimates.

**Figure 1.**
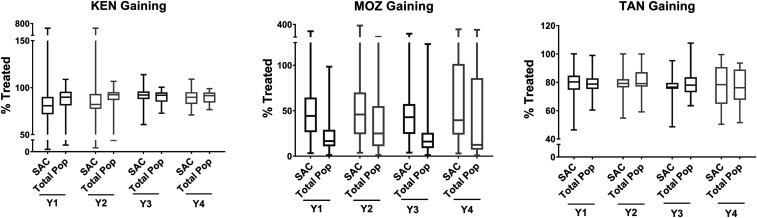
Annual study-wide coverage (% treated; median and range) for Schistosomiasis Consortium for Operational Research and Evaluation (SCORE) gaining control studies, by study. Results are presented for school-age children (SAC) and the total population (Total Pop). The boxes extend from the 25^th^ to the 75^th^ percentile. The horizontal lines within the boxes indicate the median. Whiskers extend from the smallest value to the largest. KEN = Kenya; MOZ = Mozambique; TAN = Tanzania; Y = study year.

**Figure 2.**
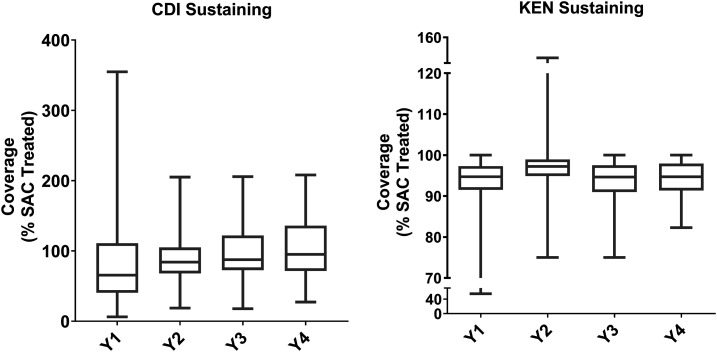
Annual study-wide coverage (% school-age children treated; median and range) for Schistosomiasis Consortium for Operational Research and Evaluation (SCORE) sustaining control studies, by study. The boxes extend from the 25^th^ to the 75^th^ percentile. The horizontal lines within the boxes indicate the median. Whiskers extend from the smallest value to the largest. CDI = Côte d’Ivoire; KEN = Kenya; SAC = school-age children; Y = study year.

**Figure 3.**
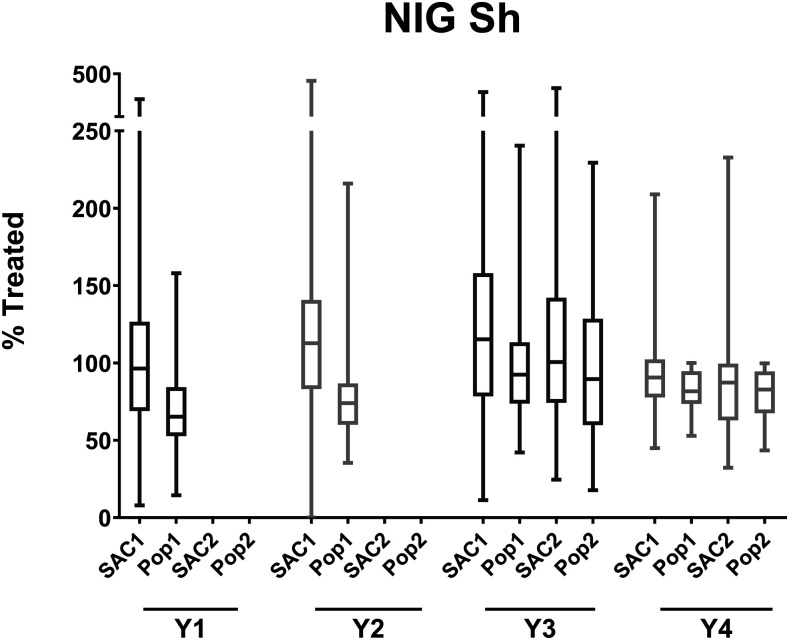
Annual study-wide coverage (median and range) for the Niger study. The boxes extend from the 25^th^ to the 75^th^ percentile. The horizontal lines within the boxes indicate the median. Whiskers extend from the smallest value to the largest. Pop1 = total population coverage in arms receiving treatment in years 1 and 2, and once-a-year treatment in years 3 and 4; Pop2 = total population coverage in arms receiving twice-a-year treatment in years 3 and 4; SAC1 = school-age children coverage in arms receiving treatment in years 1 and 2, and once-a-year treatment in years 3 and 4; SAC2 = school-age children coverage in arms receiving twice-a-year treatment in years 3 and 4.

Boxplots illustrating coverage estimates from the gaining and sustaining control studies are shown in Figures 1 and 2, respectively, and for the Niger study in Figure 3. Median coverage and ranges for all studies, stratified by year, are shown in Supplemental Table S1. Except in Mozambique, by year 4, the average overall coverage in all arms of all the studies (calculated as the average of medians across all study years) was at least 75% both for SAC and, if relevant, for the total population. Coverage among SAC was at least 90% in Côte d’Ivoire and Kenya’s sustaining control studies, the SBT arm of Kenya’s gaining control study, and two arms of the Niger study.

**Figure 4.**
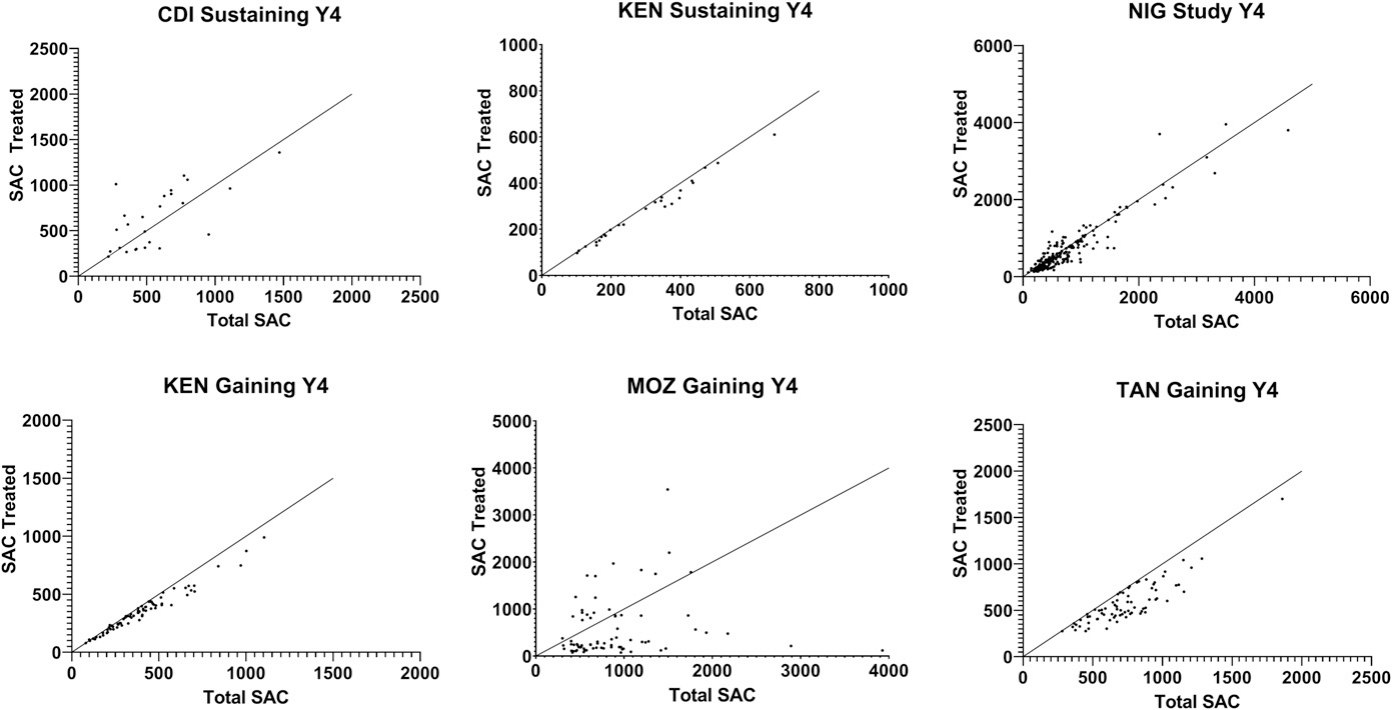
Mass drug administration (MDA) coverage in Schistosomiasis Consortium for Operational Research and Evaluation (SCORE) studies of gaining and sustaining control of schistosomiasis; comparison of eligible school-age children (SAC) and number treated during the last MDA for each study. Each point represents an individual study village. The *x* axis shows the total number of SAC and the *y* axis shows the number of SAC treated (SAC?). Note that the range of the *y* axis is different for different studies. Diagonal line represents 100% coverage. CDI = Côte d’Ivoire; KEN = Kenya; MOZ = Mozambique; NIG = Niger; TAN = Tanzania.

Village-level coverage varied substantially in some studies, especially in the early years. Figure 4 is a plot of numbers of children treated versus SAC population during the last MDA in each village in each study. By the final study MDA, some studies—such as the Kenya gaining and Kenya sustaining studies—were achieving good coverage in most villages. In others, such as in Côte d’Ivoire, the median coverage for all villages was 95% (Supplemental Table S1), but that masks a range of coverage levels that are both well below and well above 100%.

In Mozambique, the coverage range was very large, but the median coverage was low. For example, in the SBT arm in year 4, coverage among SAC was 26%, with a range of 3–79% (Figure 1, Supplemental Table S1). Coverage in individual villages also fluctuated substantially over time. In Côte d’Ivoire, the village with the greatest fluctuation in coverage between the first and last MDA reported 214% coverage in the first MDA and 78% in year 4. This was due both to an increase in the reported denominator, from 635 to 766 children, and a decrease in SAC reported treated, from 1,360 to 597. In some villages, denominators fluctuated markedly over the course of the study. For example, in one village in Niger, the numbers of SAC were reported as 217, 226, 169, and 388 in years 1, 2, 3, and 4, respectively. Numbers treated in this village did not bear a consistent relationship to denominators; coverage was reported at over 100% in years 2 and 3 and 75% and 85% in years 1 and 4, respectively.

Many studies reported individual village coverage rates well over 100%. The highest coverage rates recorded were in a CWT village in Kenya with 694% of SAC reportedly treated and an SBT village in Niger at 459%. Anecdotal reports indicated that coverage greater than 100% may have resulted from treatment being given to individuals from nearby villages who heard about the MDA, for example, through radio announcements, or inclusion of nonresident relatives of CDDs in the MDA. In some places, underestimated denominators appeared to contribute to artificially high coverage estimates.^17^

Coverage in Mozambique was the lowest among the SCORE gaining and sustaining control studies. The median SAC coverage was as low as 26% in year 4 SBT villages. One reason for the low coverage was poor school attendance. An assessment of 61 SCORE study villages by the Mozambique team in 2014 found that only 32% of children were in school. In 2015, a more comprehensive analysis among 132 SCORE study villages in all districts indicated only 35% school attendance. Challenges included employment of children in mining and other local industries and most of the community leaving villages during harvest times.

Some of the variability in coverage occurred from factors outside of a study’s control. For example, in Côte d’Ivoire, at the onset of the study, research and treatment teams had difficulty reaching some of the villages because of the fragile sociopolitical context and serious security issues.^19^ In some years, flooding in Mozambique and Niger made access to some villages extremely difficult. In Kenya and Tanzania, drought and resultant food shortages impacted willingness of some people to ingest PZQ, which can cause gastrointestinal discomfort if taken without food.^20^

### Impact of SBT versus CWT on SAC coverage.

Based on a systematic review of previous studies,^21^ we expected that SAC coverage would be highest in CWT villages. In fact, in Tanzania, SAC coverage was similar in SBT and CWT villages (Supplemental Table S1). In Kenya, after addition of schools as venues to CWT arms during years 3 and 4, coverage of children in CWT and SBT arms was also similar. Only in Mozambique, where coverage was low throughout the study and school attendance was extremely poor, was SAC coverage consistently higher in CWT villages than in SBT villages (Supplemental Table S1). In Niger, where SAC coverage tended to be above 100%, SAC coverage was generally higher in CWT villages (Supplemental Table S2).

Coverage data did not support the hypothesis that PZQ drug holidays would reduce community awareness and acceptance of MDA the year after the holiday. However, MDA with albendazole or other drugs often occurred in years when PZQ was not administered, and that could have mitigated any impact of skipping years of MDA with PZQ. In Niger, coverage was similar among those receiving annual and those receiving biannual treatment (Supplemental Table S2), suggesting that “treatment fatigue” was not an issue.

### Impact of concerted efforts to improve coverage.

In year 4, a concerted effort was made to increase CWT coverage in Mozambique, including allocating staff for CWT based on village size instead of providing a fixed and equal number to all villages, increasing CDD supervision, and providing T-shirts to CDDs conducting CWT.

**Figure 5.**
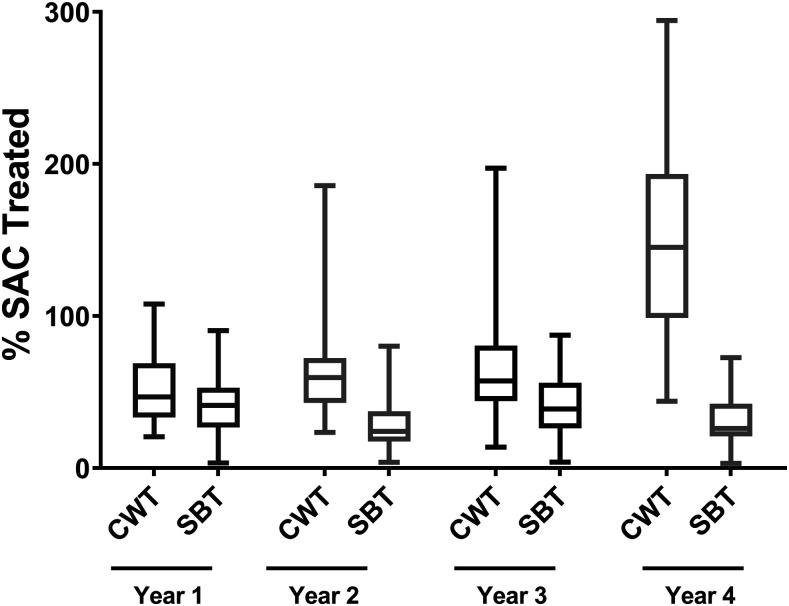
Annual school-age children coverage in villages treated with annual community-wide treatment and with annual school-based treatment in Mozambique, stratified by year. The boxes extend from the 25^th^ to the 75^th^ percentile. The horizontal lines within the boxes indicate the median. Whiskers extend from the smallest value to the largest. CWT = community-wide treatment; SBT = school-based treatment.

The impact of these interventions is illustrated in Figure 5, which shows data from the two study arms that had annual coverage data (i.e., they had no PZQ holiday years). These CDD-involved changes in implementation had little impact on SAC coverage in the study arm receiving SBT, likely due to continued very low school attendance. However, in the study arm receiving annual CWT, SAC coverage increased from less than 55% in each of the first three study years to 146% in year 4.4.

In Côte d’Ivoire’s sustaining control study and Kenya’s gaining control study, coverage improved after the first or second year. Reasons for improvement in Côte d’Ivoire included having community health workers treat non-enrolled SAC in the community to complement treatment in schools, a more stable sociopolitical context and enhanced security, and better coordination between the research team and the national control program, including having study staff participate in the MDAs, which were implemented by the Ministry of Health. In Kenya’s gaining control study, schools were included as venues for CWT starting in year 3, resulting in increased SAC coverage. On the other hand, in Tanzania, adding schools as venues for CWT in years 3 and 4 had no impact on SAC coverage in CWT villages, likely because the study in Tanzania was meeting SAC targets in CWT villages in years 1 and 2 through CDD efforts alone.

### Kenya gaining control coverage survey and follow-up.

In the second year of the Kenya gaining control study, the research team conducted a coverage survey in all 75 villages receiving CWT to assess the quality of treatment coverage data reported by the CDDs. The list of households in each village was obtained from the year 1 population census conducted by community health workers, who also served as CDDs. Depending on village size, between 15 and 30 households were selected for participation in the coverage survey. Treatment coverage was calculated as the proportion of eligible individuals who reported receiving PZQ.^17^ The survey indicated that CDD reports overestimated coverage (e.g., 84% versus 62% total population coverage from CDD reports and the coverage survey, respectively). Community drug distributors may have treated people not in the community or inflated numbers of treated individuals. In addition, during the survey it was found that CDDs had not always been diligent in conducting the year 1 census, resulting in underestimates of the eligible population and higher reported coverage than had been achieved. In follow-up, additional training was provided to CDDs before MDA in years 3 and 4. Household coverage surveys in these years indicated coverage levels closer to those reported by CDDs.

## DISCUSSION

In designing research studies, investigators often need to choose between narrowly focused, highly controlled studies that can provide clear, explanatory answers, or pragmatic studies that allow for real-world variation and may better demonstrate how an intervention will perform in practice. During the design of the gaining and sustaining control studies, SCORE had extensive input from researchers, program managers, and representatives of the WHO about how best to balance these trade-offs in the context of our charge from the Bill & Melinda Gates Foundation funders to provide quality data to help program managers make evidence-based decisions about the diagnosis, treatment, control, and elimination of schistosomiasis. The SCORE gaining and sustaining control studies implemented a design that took the middle road—conducting studies that were designed as cluster randomized field trials where the interventions and outcome measurements were performed in the context of existing national programs.^9^

An issue that became apparent during the conduct of these studies is the inherent tension between the goals of MDA programs—to treat as many people as possible—and the research, which required treatment of defined populations and minimizing the potential for “contamination,” for example, by giving PZQ to people in villages on treatment holiday who lived near villages receiving CWT. Mitigating this required working with CDDs and sensitization efforts that encouraged some people to be treated and reassured others about not being treated. This would usually not be an issue during normal NTD program operations because NTD programs normally use administrative boundaries to define MDA implementation. Thus, neighboring villages receiving different treatments would typically belong to different administrative units, which routinely might receive different interventions. Future studies using cluster randomized designs in the context of ongoing programs should pay attention to this issue of potential contamination from the start of the study to increase the likelihood of being able to measure differences between study arms.

The balance between specifying interventions and measurements in detail in multisite studies, versus providing flexibility for local context, was particularly complex for the SCORE studies. We chose not to specify aspects that we believed were routine for MDA teams and were likely to be conducted differently in different places, such as community sensitization. Details of CDD training and supervision were also left to individual study sites. During the study design, we considered requiring systematic data collection on process measures, for example, related to sensitization and CDD training, and coverage surveys. However, these ideas were discarded as too burdensome.

In Kenya’s gaining control study, a coverage survey in year 2 demonstrated problems with CDD data, similar to those reported in other evaluations of NTD coverage.^22,23^ Subsequently, in Kenya, in year 3, supervision of CDDs serving study areas was increased. Changes in CDD supervision and workloads contributed to a measurable impact in the number of individuals treated in Mozambique in year 4 (Figure 5). Future studies may want to be more prescriptive and collect more process-related data about how well interventions are being implemented.

### Achieving coverage targets.

The SCORE gaining and sustaining control studies resulted in several suggestions for improving coverage, as described in a SCORE publication from Kenya^24^ and identified in other studies and a SCORE-supported systematic review.^21^ These include using schools as venues in community-wide efforts (as was required in SCORE CWT villages), community education addressing topics such as the need for MDA despite the absence of overt symptoms of disease, providing food during MDA, and providing incentives for CDDs and teachers.

The lowest coverage rates were in Mozambique—as low as 3% in some villages. Contributing factors to these low rates included cholera outbreaks, flooding, political unrest, children and adults working some distance away from the village, and only limited sensitization. Some of the problems we encountered in Mozambique and other countries, for example, inaccessibility of villages due to flooding when MDA was supposed to occur, could not be easily addressed through study modifications. Other events could have been anticipated and mitigation strategies developed. For example, MDAs could have been planned to avoid annual times of food shortages or food could have been included with MDA. Of note, including food has the potential added benefits of increasing participation, decreasing adverse events, and increasing bioavailability.^20,25^ To the extent possible, future studies should anticipate issues and design consistent and systematic ways to address them, rather than the ad hoc approach taken in this multicountry assessment.

In some study villages, PZQ treatments beyond those called for in the protocol were provided. For example, in Tanzania, in year 4, four villages meant to be on treatment holiday mistakenly received MDA. In Kenya’s sustaining control study in year 4, two different private organizations treated the same village, resulting in two PZQ MDAs in 1 year, and one that should have been on holiday was treated. Many countries are struggling with how to organize the efforts of multiple implementing partners to improve efficiency and reduce the kinds of issues we experienced, not only for schistosomiasis but also for a range of NTDs addressed through preventive chemotherapy. The potential for a mismatch between the planned treatment and what is actually delivered may be particularly difficult in a randomized trial, where nearby villages in one administrative district may be receiving different regimens, but it is also an ongoing programmatic issue. National leadership and coordination with national and international partners to address this issue is critical.

During the development of the harmonized study protocol, there was concern that extra sensitization might be required the year after a village experienced a PZQ drug holiday, that is, the year following a year when no PZQ treatment was provided. This could potentially offset some of the economic savings achieved by skipping an MDA. However, perhaps because villages were receiving multiple health campaigns, PZQ drug holiday years did not seem to impact subsequent year coverage.^18^ In fact, communities on drug treatment holiday sometimes were concerned that they were not receiving treatments when neighboring villages were, requiring additional communication investments to assuage concerns about skipping treatment during drug holiday years. There was also concern that villages receiving twice-yearly treatment might experience “treatment fatigue.” In Niger, coverage was similar among those receiving annual and those receiving biannual treatment (Supplemental Table S2).

Although the protocol called for treatment of pregnant women, consistent with the WHO guidelines indicating that such treatment is safe,^26,27^ women known to be pregnant were excluded from MDA in CWT villages in Tanzania. Authorities expressed concern over lack of local data confirming safety. Addressing this issue may require working with high levels of government to change policies and locally with the CDDs to ensure their buy-in and improve acceptance at the village level.

### Measuring coverage.

The difficulty in developing good coverage estimates has been well-documented.^5–7,28^ In planning the SCORE studies, annual coverage surveys were considered, but ultimately were not required by the harmonized study protocol because of lack of consensus on how to conduct coverage surveys and concerns about the financial and human resources that would be required. Instead, it was deemed acceptable to collect data on the numbers treated and compare those with the population eligible for treatment. Whereas some studies conducted coverage surveys, for example, the Kenya gaining control study, most did not.

After SCORE was designed, a multicountry feasibility study (Burkina Faso, Honduras, Malawi, and Uganda) was conducted by the Ministries of Health in these countries, the WHO, and the NTD Support Center of the Task Force for Global Health. In 2016, the WHO Strategic and Technical Advisory Group on NTDs endorsed the use of probability sampling and segmentation as the preferred method for evaluation of coverage of preventive chemotherapy, and the WHO developed a set of training protocols and analysis tools to support countries to perform these coverage surveys.^29,30^

In retrospect, investment in coverage surveys would likely have produced much better data on coverage. Whether they would have improved coverage in places like Mozambique, where coverage was very low for the most part, is unclear, as most studies did not use their data to guide post-MDA mop-up activities or to modify study-wide approaches. Nevertheless, given the importance of collecting and using coverage data in study implementation and analysis, and also that the WHO guidance on how to conduct coverage surveys is now available,^29,30^ future longitudinal studies involving MDA should strongly consider supporting formal coverage surveys.

As others have found,^22,23,28^ CDD reports of numbers treated are often not ideal for estimating coverage. The many CWT villages with coverage rates well over 100% illustrate the importance of having CDDs record whether people who are given drugs are from the study village. It is also important to ensure that CDDs understand where the study considers village boundaries to lie, as definitions that are widely accepted in the community or may apply during non-research efforts may differ from the government-defined boundaries being used to define villages in a study. Sometimes, the definitions the CDDs used for the population requiring treatment were based on tribal affiliations, so minority populations were not included.^31^ In addition to the issues related to how CDDs defined villages, administrative changes and building of new schools also impacted both numerators and denominators. Future studies should consider systematically capturing information on these changes and deciding in advance how to address them.

Denominator data were particularly problematic. Ideally, each SCORE study would have conducted at least one census during the study period. Because resources and capacity were limited, the protocol did allow investigators to use available community/village data for denominators, if they were of high quality. In the Tanzania and Kenya gaining control studies, population estimates were based on data collected at the same time as the SCORE study and were probably adequate. For SBT, in countries where school registration was high, such as Kenya and Tanzania, school registries were usually used for denominators. However, because schools sometimes enrolled children from other villages and because there was village-level variability in accuracy of enrollment data, we cannot know whether these provided a good measure of the SAC in any given village. In Côte d’Ivoire and Niger, Ministry of Health data were assumed to be of high quality. However, on use, substantial concerns were raised on the reliability of these data because of frequent occurrences of very high coverage estimates and large unexplained variations from year-to-year in some villages. Despite repeated efforts, further specific information about how many village-level population estimates were developed remained unavailable.

In Mozambique, where a census was conducted at the beginning of the study, dramatic population shifts occurred during the study, and there were no reliable data on which to make village-level population adjustments. In year 5, investigators attempted to use an innovative modeling approach using data from the Facebook Connectivity Lab project. They combined these data with data from a 30-village census to estimate numbers of individuals, and of SAC, per building identifiable by Google Earth imagery in the studied villages. However, applying the estimates of total population per structure and SAC per structure to other villages in the Mozambique study resulted in considerable variability in village population estimates, which did not appear superior to the previous estimates based on the 2011 census. Because the new estimates could not be validated, the decision was made to use the 2011 census data as the denominators for all years of the study. Although the year 3 attempt to model the population using Facebook Connectivity Lab data was not successful, modeling of populations using remote sensing and artificial intelligence-based tools may be an option in some cases and is likely to be more useful as tools improve.

In addition to these intra-country variations, which affected numerators, denominators, and, ultimately, coverage estimates in the studies, the inter-country variations in the records from which the denominators were sourced meant that comparisons in results were not like-for-like across the studies.

### Future studies of schistosomiasis control using MDA.

We appreciate the efforts of the SCORE study teams to ensure high-quality MDA and coverage data. Nevertheless, the quality concerns that SCORE experienced limited the use of these data in analyses to help explain the parasitologic study outcomes. Studies will continue to evaluate the use of MDA for control and elimination of schistosomiasis, both alone and in concert with other interventions. In all of these, providing high-coverage MDA will be essential, and accurately measuring coverage will help researchers better understand their study outcomes. Future studies should consider planning for issues such as those we encountered, establishing systems for timely collection of information about what actually happened during study implementation, including coverage surveys, and providing adequate resources for early identification and correction of issues that could result in low coverage or inaccurate coverage measurement.

## Supplemental tables

Supplemental materials
