# Supplementary material for: Lessons Learned in Conducting Mass Drug Administration for Schistosomiasis Control and Measuring Coverage in an Operational Research Setting
Source: Am J Trop Med Hyg. 2020 May 12;103(1 Suppl):105–13. doi: 10.4269/ajtmh.19-0789 (PMC7351302; doi:10.4269/ajtmh.19-0789)
Supplement: Supplementary file 1 [file tpmd190789.SD1.pdf]

Supplemental Table S1. Estimated annual study-wide coverage (median and range) for SCORE Gaining and Sustaining Control Studies, stratified by study. Overall coverage was calculated as the average of the medians across all years that villages received treatment.

SCORE Study	Type of MDA / Population Assessed	Year 1 Median % (Range)	Year 2 Median % (Range)	Year 3 Median % (Range)	Year 4 Median % (Range)	Overall % Coverage
**Côte d'Ivoire Sustaining Control**	SBT SAC	66 (6, 355)	84 (19, 205)	88 (18, 206)	95 (27, 208)	83
**Kenya Sustaining Control**	SBT SAC	95 (55, 100)	97 (75, 132)	95 (75, 100)	95 (82, 100)	95
**Kenya Gaining Control**	SBT SAC	85 (59, 103)	88 (75, 125)	93 (62, 114)	91 (74, 109)	90
	CWT SAC	72 (9, 694)	79 (15, 693)	88 (61, 101)	87 (71, 99)	83
	CWT total population	90 (25, 109)	92 (44, 107)	92 (73, 101)	91 (77, 99)	91
**Mozambique Gaining Control**	SBT SAC	41 (3, 132)	33 (4, 375)	32 (4, 95)	26 (3, 79)	33
	CWT SAC	51 (4, 252)	55 (13, 186)	45 (14, 197)	145 (44, 294)	52

	CWT total population	29 (5, 98)	43 (6, 130)	53 (18, 123)	120 (74, 283)	26
**Tanzania Gaining Control**	SBT SAC	81 (73, 100)	80 (68, 100)	77 (49, 95)	84 (50, 100)	81
	CWT SAC	77 (47, 99)	78 (55, 100)	76 (56, 83)	76 (60, 100)	77
	CWT total population	79 (60, 99)	79 (59, 100)	78 (63, 108)	76 (52, 94)	78

Supplemental Table S2. Estimated study-wide coverage (median and range) in the Niger study, by year and by year 3 randomization to once-a-year vs. twice-a-year MDA. Overall coverage estimates are calculated as the average of the medians across all treatment years.

Type of MDA/ Population Assessed	Year 1 Median % (Range)	Year 2 Median % (Range)	MDA Protocol for Years 3 and 4		Year 3 Median % (Range)	Year 4 Median % (Range)	Overall Coverage
SBT (SAC only)	90	103	Once-a-year		115	87	101
	(8, 352)	(0, 362)			(15, 335)	(53, 102)	
			Twice-a-year	(1st)	119	85	102
					(11, 394)	(45, 105)	
				(2nd)	94	78	86
					(25, 416)	(32, 100)	
CWT SAC	108	120	Once-a-year		108	111	110
	(45, 330)	(37, 459)			(56, 333)	(57, 167)	
			Twice-a-year	(1st)	130	124	127
					(38, 345)	(56, 209)	
				(2nd)	133	110	121
					(40, 296)	(48, 233)	
CWT total population	65	74	Once-a-year		90	84	87
	(14, 158)	(36, 159)			(18, 229)	(61, 99)	
			Twice-a-year	(1st)	96	81	89

		(42, 241)	(53, 100)	
	(2nd)	90	83	86
		(18, 229)	(43, 100)	
